# The Macronutrient Composition of Infant Formula Produces Differences in Gut Microbiota Maturation That Associate with Weight Gain Velocity and Weight Status

**DOI:** 10.3390/nu14061241

**Published:** 2022-03-15

**Authors:** Julie A. Mennella, Yun Li, Kyle Bittinger, Elliot S. Friedman, Chunyu Zhao, Hongzhe Li, Gary D. Wu, Jillian C. Trabulsi

**Affiliations:** 1 Monell Chemical Senses Center, Philadelphia, PA 19104, USA; mennella@monell.org; 2 Department of Biostatistics, Epidemiology and Informatics, Perelman School of Medicine, University of Pennsylvania, Philadelphia, PA 19104, USA; yunli1@pennmedicine.upenn.edu (Y.L.); hongzhe@pennmedicine.upenn.edu (H.L.); 3 Division of Gastroenterology, Hepatology, and Nutrition, Children’s Hospital of Philadelphia, Philadelphia, PA 19104, USA; bittingerk@chop.edu (K.B.); chunyu.zhao@czbiohub.org (C.Z.); 4 Division of Gastroenterology and Hepatology, Perelman School of Medicine, University of Pennsylvania, Philadelphia, PA 19104, USA; elliotf@pennmedicine.upenn.edu (E.S.F.); gdwu@pennmedicine.upenn.edu (G.D.W.); 5 Department of Behavioral Health and Nutrition, University of Delaware, Newark, DE 19713, USA

**Keywords:** infant, microbiota, metabolome, diet, gene, infant formula, rapid weight gain, randomized controlled trial

## Abstract

This proof-of-principle study analyzed fecal samples from 30 infants who participated in a randomized controlled trial on the effects of the macronutrient composition of infant formula on growth and energy balance. In that study, infants randomized to be fed cow milk formula (CMF) had faster weight-gain velocity during the first 4 months and higher weight-for-length Z scores up to 11.5 months than those randomized to an isocaloric extensive protein hydrolysate formula (EHF). Here we examined associations among infant formula composition, gut microbial composition and maturation, and children’s weight status. Fecal samples collected before and monthly up to 4.5 months after randomization were analyzed by shotgun metagenomic sequencing and targeted metabolomics. The EHF group had faster maturation of gut microbiota than the CMF group, and increased alpha diversity driven by Clostridia taxa. Abundance of *Ruminococcus gnavus* distinguished the two groups after exclusive feeding of the assigned formula for 3 months. Abundance of Clostridia at 3–4 months negatively correlated with prior weight-gain velocity and body weight phenotypes when they became toddlers. Macronutrient differences between the formulas likely led to the observed divergence in gut microbiota composition that was associated with differences in transient rapid weight gain, a well-established predictor of childhood obesity and other comorbidities.

## 1. Introduction

There are sensitive periods during early life when diet interacts with the gut microbiota in shaping the structure and function of the mucosal immune system and, in turn, modifying metabolic and immune health [1,2,3]. While the initial colonization pattern of newborns’ gut microbiome is often considered chaotic, with low species diversity, a growing body of evidence—the vast majority from observational and cross-sectional research—suggests that maturation and diversification of the gut bacterial environment during infancy is shaped by early diet [4,5,6,7,8,9].

Although the early diet is unique in typically consisting of a sole source of nutrition in liquid form—breast milk or breast milk substitute—the type of diet is important in shaping the gut microbiome. During the first 4 months, gut microbial alpha diversity of exclusively breastfed infants, as expressed by the standardized Shannon index, was consistently lower than in breastfed infants whose diets were supplemented with infant formula and/or solid foods [9,10]. Supplementation with relatively small amounts of infant formula can shift the more “stable” and uniform microbiome of exclusively breastfed infants to one with a broader and more diversified spectrum of bacteria [8,11,12]. Prominence of Enterobacteriaceae, especially *E. coli* and *K. pneumoniae*, and abundance of Clostridiaceae have been observed among formula-fed infants. In particular, the relative abundance of Clostridia, a class of bacteria involved in amino acid fermentation [13,14,15], is greater in formula-fed than in breastfed infants younger than 6 months [5,11,16].

Based on widespread evidence that diet plays a significant role in the composition of the gut microbiota and long-term health, we hypothesized that there would be significant differences in the gut microbiome over time in infants exclusively fed different infant formulas that are isocaloric yet with different macronutrient composition. Specifically, we posited differences between infants fed cow milk formula (CMF), the most commonly ingested type of infant formula [17], and infants fed an extensive protein hydrolysate formula (EHF), a type of formula for infants with intolerance to cow’s milk proteins. Unlike the intact proteins found in CMF, the proteins in EHF have been hydrolyzed to reduce the burden of digestion and allergenicity; consequently, EHF is abundant in free amino acids and small peptides [18]. While the carbohydrate source in CMF is lactose, EHF carbohydrate comprises non-lactose sources that, along with free amino acids, have been shown to impact the composition and function of the adult gut microbiome [19,20]. Randomized controlled trials (RCTs) have consistently revealed that infants fed EHF exhibit more normative weight gain during early infancy [21,22,23] and decreased risks of allergic diseases during childhood when compared to those fed CMF. For example, in the German Infant Nutritional Intervention RCT, breastfed infants with a family history of atopy who were randomized at birth to be fed EHF, only if human milk was insufficient, remained at decreased risk for atopy 20 years later compared to those randomized to CMF [24].

In the present proof-of-principle study, we randomly selected a subsample of 30 infants from our RCT of exclusively formula-fed infants with no family history of atopy. The RCT demonstrated that the accelerated weight gain pattern among CMF-fed infants compared to EHF-fed infants was due to both energy intake and energy loss mechanisms. In this subsample, we focused on group-dependent differences in the maturation of the microbiota from 0.5 to 4.5 months, when formula feeding was exclusive and when the microbiota is highly plastic and responsive to external factors, such as diet [25]. We posited that there would be time-dependent differences in gut microbiome composition that were related to differences in the macronutrient composition of the randomized formulas that would, in turn, be associated with clinical outcomes. We used an integrated analysis of shotgun metagenomic sequencing and metabolomics on fecal samples collected before (baseline: 0.5 and 0.75 months) and at fixed intervals after randomization (1.5, 2.5, 3.5, and 4.5 months) to determine the effects of infant formula diet on the composition and metabolic function of the infant gut microbiome and its relationship to clinical outcomes.

## 2. Materials and Methods

### 2.1. Participants and Trial Design

Study participants were enrolled in a longitudinal, double-blind RCT on healthy, term infants designed to determine the effects of feeding isocaloric commercial infant formulas (CMF, EHF) that differed in macronutrient composition (see Section 2.1.2) on early weight gain and energy balance. From the intent-to-treat cohort of 113 infants, we randomly selected 30 infants while balancing for sex and race/ethnicity. The study design, inclusion and exclusion criteria, and CONSORT table for the 12-month-long RCT have been published previously [26]. In brief, infants who were born at term and whose mothers chose to exclusively formula feed when infants were 2 weeks of age were randomized to be fed either CMF or EHF for the first year. The Office of Regulatory Affairs at the University of Pennsylvania approved the study, and the trial was registered online at clinicaltrials.gov (NCT01700205) prior to its start.

#### 2.1.1. Study Cohort

The cohort of 30 exclusively formula-fed infants (15 CMF, 15 EHF) was racially diverse (57% black, 27% white, 17% more than one race). There were no significant differences between the formula treatment groups in sex ratio of infants or any baseline infant or maternal characteristics measured [26], including infant anthropometric measurements taken at baseline (Table 1).

#### 2.1.2. Composition of Infant Formula Diets

The two infant formulas (Enfamil [CMF], Nutramigen [EHF]; Mead Johnson Nutrition) were isocaloric (67 kcal/100 mL) and contained no added prebiotics or probiotics. While the percent energy provided from fat (48% [5.3 g/100 kcal]) and type of fat blend (i.e., palm olein, soy, coconut, high-oleic sunflower oils) were similar, the percent energy from carbohydrate (EHF: 41% [10.3 g/100 kcal], CMF: 44% [11.3 g/100 kcal]) and the percent energy from protein (EHF: 11% [2.8 g/100 kcal], CMF: 8% [2.1 g/100 kcal]) slightly differed [27]. The major differentiators were the form of the protein and the source of carbohydrate. CMF contained intact cow milk proteins and its source of carbohydrate was lactose, whereas EHF contained small peptides [28] and substantially higher concentrations of free amino acids (80,375 µmol/L) because of its extensive hydrolyzation than CMF (864 µmol/L) [18], and its source of carbohydrate was primarily corn syrup solids with some modified cornstarch.

#### 2.1.3. Clinical Phenotypes

We tested measures of anthropometry and body composition from the RCT [26] for associations with infants’ microbiota identified in this proof-of-concept study. At baseline visits (0.5, 0.75 months) and each month thereafter until 12.5 months, infants were weighed and measured in triplicate by research personnel certified in standard anthropometric techniques and with a calibrated pediatric scale (Scale-Tronix, White Plains, NY, USA) and an infantometer (Harpenden Infantometer 702; Crymych, Dyfed, UK) that were accurate to 0.001 kg and 0.1 cm, respectively. Anthropometric data were converted to weight-for length Z (WLZ) and length-for age Z scores using World Health Organization growth standards [29]. Velocities of early weight (g/day) and length (cm/day) accretion were calculated by dividing the change of weight in grams or length in centimeters by the change of age in days from 0.5 to 4.5 months, when infant formula accounted for 93% (±3) of daily energy intake. Of the 30 children in this cohort, 26 completed the final RCT visit at 12.5 months. At this age, only 24% (±5) of their daily energy intake was from infant formula. We determined children’s weight status at 1 year based on age- and sex-specific WLZ percentiles, which defines overweight as values greater than the 85th percentile [30]. There was no difference between the groups in the age at which mothers began complementing their diets with solids (*p* = 0.56).

Measures of body composition were obtained by the double-labeled water method at the baseline visit and at 3.5 and 12.5 months [26,31]. After obtaining a baseline urine sample, infants were dosed based on body weight with 0.3 g H_2_^18^O and 0.15 g ^2^H_2_O per kilogram estimated total body water (TBW). Mothers were given special study diapers to collect morning urine samples each day thereafter for one week. The elimination rates of ^18^O and ^2^H were used to determine isotope dilution spaces (kg) and TBW, from which we determined fat-free mass (kg) for each infant at each age by dividing TBW derived from ^18^O and ^2^H dilution by age-specific hydration constants [32,33]. From these data, we determined the fat mass (kg) and percent body fat for each infant at each age. Of the 30 infants, body composition data were available for 27 infants at 0.75 months, 28 infants at 3.5 months, and 25 infants at 12.5 months.

### 2.2. Methodology

During the trial, convenient stool samples were collected in 76 × 20 mm feces containers (Sarstedt, Inc., Newton, NC) and frozen at −70 °C until transport to the Children’s Hospital of Philadelphia Microbiome Center for analysis. For the present shotgun metagenomics and bacterial gene analyses, we analyzed 148 fecal samples. For each infant, we had at least one baseline (0.5, 0.75) sample and at least three post-randomization stool samples (1.5, 2.5, 3.5, 4.5 months); sample size at each time point ranged from 19 to 29. For the metabolomics analysis, we analyzed 137 fecal samples from 29 infants; the sample size at each time point ranged from 19 to 26. Only one baseline sample was available for the remaining infant, which we thus eliminated from this analysis. 

#### 2.2.1. Shotgun Metagenomics

The gut microbiome was characterized by shotgun metagenomic sequencing using established methods [34]. Genomic DNA was extracted using the MO BIO PowerSoil kit and prepared for sequencing with the Nextera XT kit (Illumina, Inc., San Diego, CA, USA). Libraries were sequenced on Illumina HiSeq 2500 using the paired-end 125 bp sequencing protocol. Sequence reads were processed to remove adapter sequences and low-quality reads with Trimmomatic v. 0.33 [35]. Taxonomic assignments were generated with MetaPhlAn2 [36]. Gene orthologs were assigned by aligning reads to the KEGG database [37] using DIAMOND v. 2.0.4 [38]. Negative (mock purification of DNA-free water) and positive (standard fecal and pond-sediment samples) controls were included in each assay. Samples of each formula (CMF, EHF) were assayed for DNA content, which yielded no detectable bacterial DNA. Shotgun libraries were generated from 1 ng of DNA using the NexteraXT kit (Illumina, Inc.). Libraries were sequenced on the Illumina HiSeq using 2 × 125 bp chemistry in high-output mode. 

#### 2.2.2. Targeted Metabolomics

Targeted metabolomics of 17 fecal amino acids were characterized using established methods [39] with a limit of detection of 5 nmol/g. Briefly, methanol-extracted fecal samples (5 µL/mg stool) were prepared using the Waters AccQ-Tag Ultra Amino Acid Derivatization and Chemistry Kit (Waters Corporation, Milford, MA, USA). Samples were analyzed on Waters Acquity uPLC System with an AccQ-Tag Ultra C18 1.7 μm 2.1 × 10 m column and a photodiode-array detector (Waters Corporation, Milford, MA, USA).

### 2.3. Statistical Analyses 

The abundance of genes was normalized across samples using quantile normalization (up to 95% percentile). Genes absent from more than 50% of samples were considered low-occurrence genes and were removed from the analyses. Linear mixed-effects modeling was used to determine the effect of treatment group (CMF, EHF), time (0.5, 0.75, 1.5, 2.5, 3.5, 4.5 months), and group × time interaction on (a) Shannon diversity indices, (b) relative abundance of each bacteria at the species levels, (c) the quantile normalized abundance of the top 1000 most prevalent genes, and (d) concentration of each fecal amino acid separately. The species for which more than 90% of the samples were undetected were excluded from the linear mixed effects modeling. The relative abundance of species, and the quantile normalized abundance of genes, were log10 transformed in the linear mixed effects model. A permutational multivariate analysis of variance (PERMANOVA) test on Bray–Curtis distances was used to examine differences in beta diversity between formula groups at each time point separately. A Bayesian logistic regression model was applied to determine whether specific species (using log10 transformed relative abundance in the model) were associated with formula group at each time point separately. Receiver operator characteristic (ROC) curves [40] were plotted and area under the curve values were calculated to evaluate the model. 

To probe the relationships between continuous outcomes related to gut microbiota or metabolome and formula-induced differences in clinical phenotypes reported previously [26,41], simple and partial Pearson correlation analyses were conducted. Statistical analyses of data on gut microbiota, fecal amino acids, and bacterial genes were implemented in R (version 4.1.1) and higher [42] or Graphpad Prism (version 9.2.0; Graphpad Software Inc., San Diego, CA, USA); associations with clinical outcomes were conducted using Statistica version 14.0 (StatSoft Inc., Tulsa, OK, USA). The Benjamini and Hochberg false discovery rate (FDR) [43]; *p* < 0.05 was considered significant.

## 3. Results

### 3.1. Outgrowth of Ruminococcus Gnavus and Other Clostridia Species Driven by Formula-Induced Differences in Gut Microbiota

**Figure 1:**
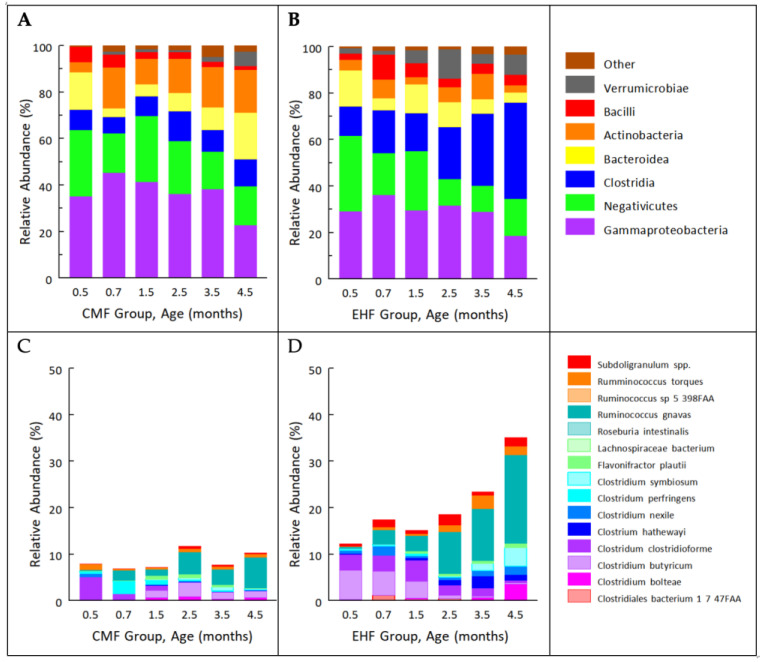
Relative abundance of microbial classes in (**A**) CMF (cow milk formula) and (**B**) EHF (extensive protein hydrolysate formula) groups and the top 15 species of Clostridia in (**C**) CMF and (**D**) EHF groups from 0.5 to 4.5 months.

The most abundant classes of bacterial taxa found in the microbiota of the 30 infants in this study, aged 0.5 to 4.5 months, were Verrucomicrobiae, Bacilli, Actinobacteria, Bacteroidia, Clostridia, Negativiutes, and Gammaproteobacteria. The EHF group had greater Shannon diversity in the gut microbiota over time than the CMF group (group × time, *p* = 0.004; linear mixed effects model). The most substantial group difference was the increased prevalence of obligately anaerobic communities of Clostridia in the EHF group (Figure 1A,B). Figure 1C,D depict the relative abundance of the top 15 Clostridia species in CMF and EHF groups, respectively. 

**Figure 2:**
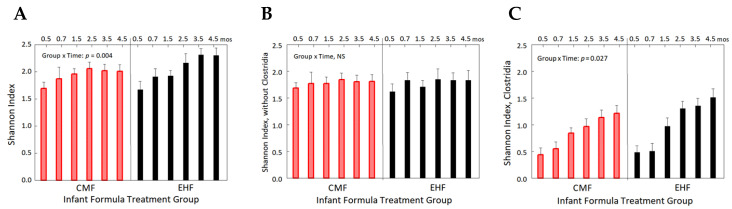
Shannon diversity indexes of (**A**) complete microbial community, (**B**) microbial community without Clostridia, and (**C**) Clostridia only. Red bars depict the CMF (cow milk formula) group and black bars depict the EHF (extensive protein hydrolysate formula) group from 0.5 to 4.5 months (linear mixed-effect modeling).

To determine whether the significant group × time interaction (*p* = 0.004) in the Shannon diversity index of the complete microbial community (Figure 2A) was due primarily to differences in Clostridia, we compared Shannon indexes of the complete community without Clostridia and to that of Clostridia only. As shown in Figure 2B, removal of Clostridia species from the Shannon diversity index resulted in no significant group, time, or group × time effect (*p* > 0.05). However, there was a significant group × time interaction in the Shannon diversity index of Clostridia only (*p* = 0.027; Figure 2C), thus providing further evidence that the greater Shannon diversity among the EHF group was driven primarily by Clostridia.

**Figure 3:**
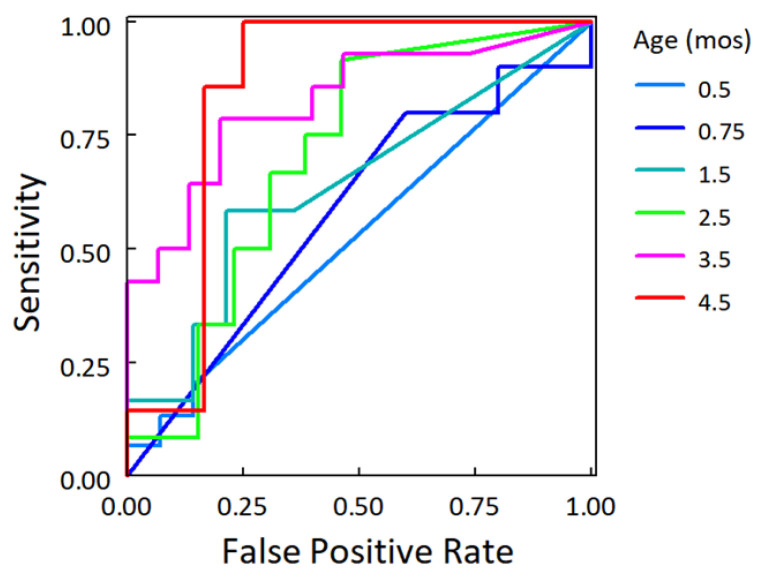
Receiver operating characteristic curves revealed discriminatory power of *Ruminococcous gnavus* to distinguish CMF group from EHF group by 3.5–4.5 months; logistic regression model.

**Figure A1:**
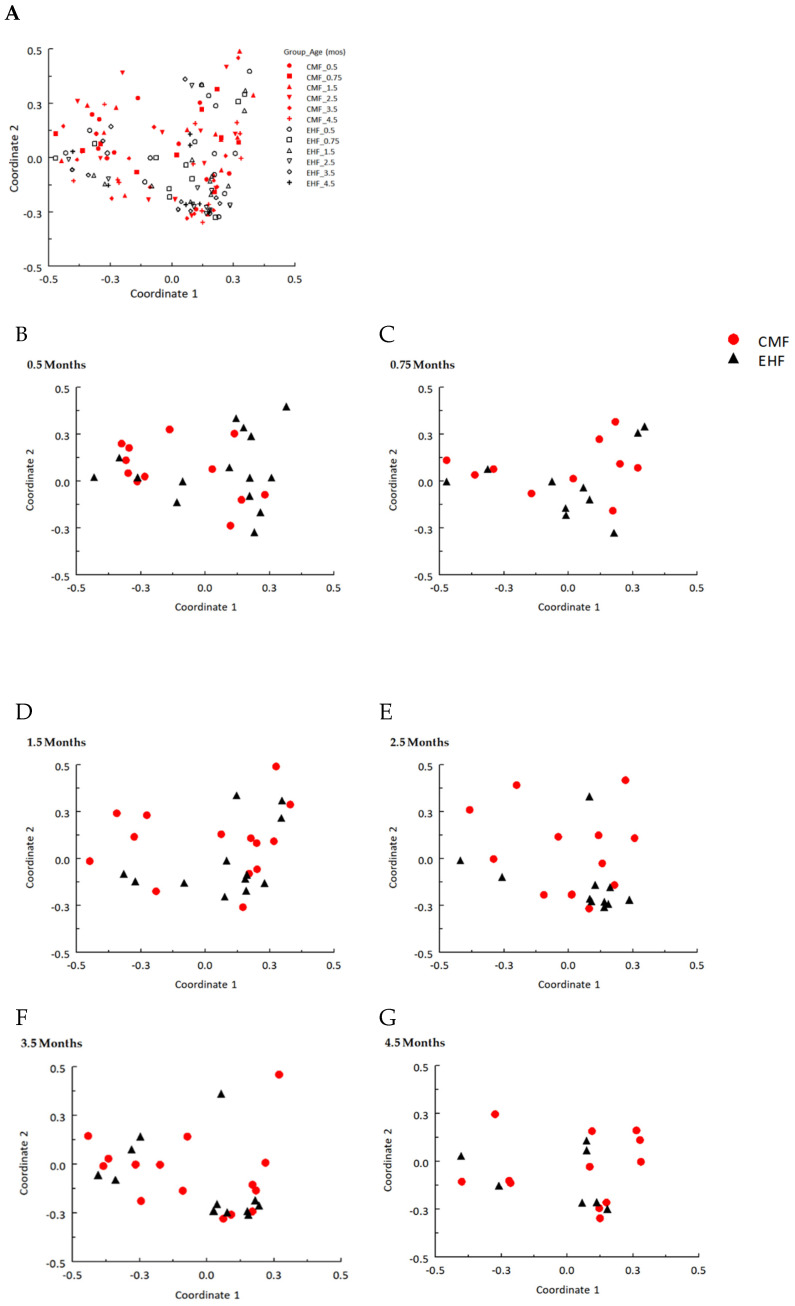
Multidimensional scaling plot of beta diversity based on Bray–Curtis distance metric for CMF and EHF groups showing (**A**) all ages combined and (**B**–**G**) at each time point. There were no significant differences between CMF (cow milk formula, red symbols) and EHF (extensive protein hydrolysate formula, black symbols) groups at 0.5, 0.75, and 1.5 months (*p* > 0.05, Permutational Multivariate Analysis of Variance (PERMANOVA) tests on Bray–Curtis distances), but there were significant group differences at 2.5 (*p* = 0.03), 3.5 (*p* = 0.02), and 4.5 months (*p* = 0.03); PERMANOVA tests on Bray–Curtis distances).

While there were no group differences before randomization to the study formulas before 1.5 months (PERMANOVA tests on Bray–Curtis distances, *p* > 0.05; Figure A1), there were significant group differences in the beta diversity index at 2.5, 3.5, and 4.5 months (*p* = 0.027, 0.024, and 0.031, respectively). By 4.5 months, the outgrowth of the most abundant taxa of Clostridia—*Ruminoccocus gnavus*—accounted for approximately one-fifth (mean relative abundance, 19.1%) of the community in the EHF group (Figure 1D), but only 6.6% in the CMF group (Figure 1C). To evaluate the predictive value of *R. gnavus* relative abundance by group, we applied logistic regression models to each time point separately (the relative abundances were log10 transformed) and constructed ROC curves (Figure 3). The area under the curve was greater than 0.8 at both 3.5 and 4.5 months, demonstrating that relative abundance of *R. gnavus* alone could distinguish the microbiome of infants fed EHF from those fed CMF after the infants had been fed the formula for approximately 3 months. 

**Figure 4:**
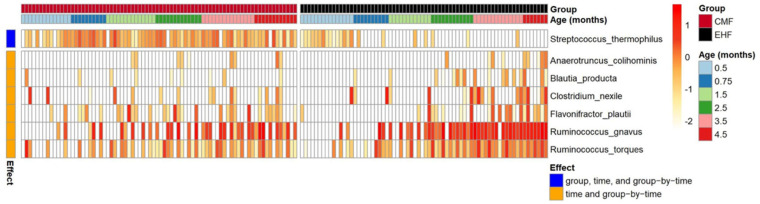
Heatmap of bacterial species from 0.5 to 4.5 months in CMF (red bar) and EHF (black bar) groups. Red signifies higher abundance. Linear mixed effects models revealed significant group × time, group and time effects (blue) for *Steptococcus thermophiles* and significant group × time and time effects (orange) for *Ruminococcus gnavus*, *Blautia producta*, *Ruminococcus torques*, *Clostridium nexile*, *Flavonifractor plautii*, *and Anaerotruncus colihominis*.

Although *R. gnavus* was the most prominent signature distinguishing the microbiome of EHF- from CMF-fed infants, other microbial taxa differed between the groups. Using a linear mixed-effect model (*p*’s < 0.05), we identified seven taxa that had significant group × time interactions (Figure 4). The taxa *Streptococcus thermophilus* had significant group × time interaction (*p* < 0.001), group (*p* < 0.001) and time (*p* = 0.005) effects with relative abundance remaining high in CMF but decreasing in the EHF group over time. Conversely, the remaining six taxa, each within the Clostridia class of Firmicutes, had significant group × time interaction (all *p*’s < 0.04) and time (all *p*’s < 0.001) effects with relative abundance being higher in EHF than CMF group. 

Table A1 lists the 47 bacterial taxa that were not affected by the randomization but significantly changed from 0.5 to 4.5 months (time effect only, *p* < 0.05). In general, some species in the Veillonella, Lactobacillus, Streptococcus genera are in higher relative abundance during the first month and then decreased over time, whereas some Bifidobacteria and Clostridium species increased over time [44,45]. 

### 3.2. Formula-Induced Differences in Expression of Genes Related to Carbohydrate Metabolism

**Figure 5:**
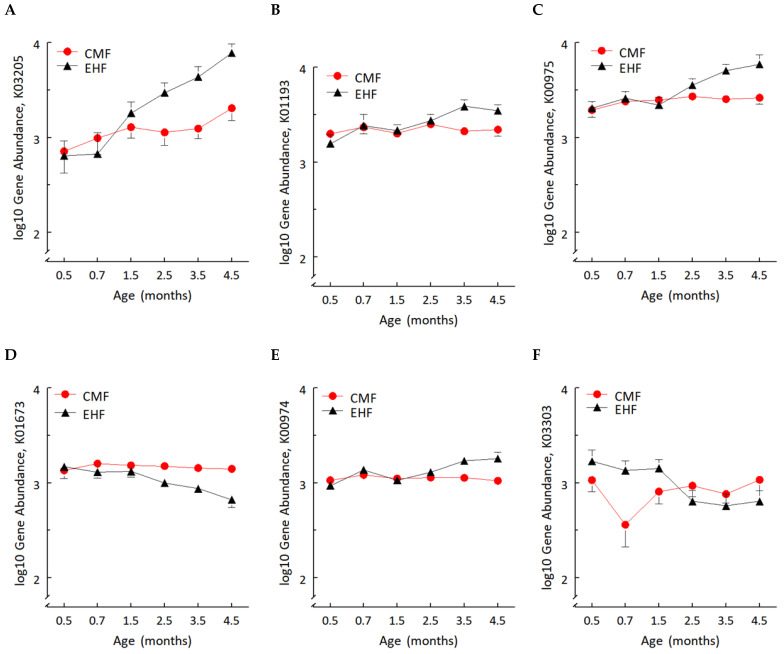
Log_10_ gene abundance over time in CMF (red circles) and EHF (black triangles) groups for gene K03205, type IV secretion system protein VirD4 (**A**); K01193, beta-fructofuranosidase (**B**); K00975, glucose 1-phosphate adenylyltransferase (**C**); K01673, carbonic anhydrase (**D**); K00974, tRNA nucleotidyltransferase (**E**); and K03303, lactase permease (**F**). Significant group × time effect for each gene; linear mixed models.

Of the top 1000 most abundant genes, we found significant group × time interactions for six genes (all FDR, *p*’s < 0.05): the type IV secretion system protein VirD4, carbonic anhydrase, tRNA nucleotidyltransferase, and three genes associated with carbohydrate metabolism: beta-fructofuranosidase, glucose-1-phosphate adenylyltransferase and lactase permease (Figure 5A–F). 

### 3.3. The Two Groups Shared Similar Fecal Amino Acid Concentrations despite Different Free Amino Acid Concentrations in CMF and EHF

**Figure 6:**
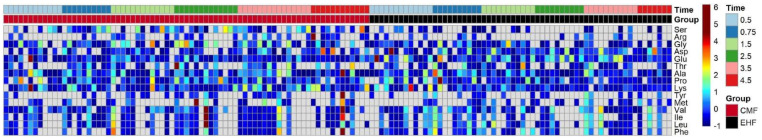
Heatmap of fecal amino acid concentrations (standardized by mean and standard deviation per amino acid) from 0.5 to 4.5 months in CMF (cow milk formula; red bar) and EHF (extensive protein hydrolysate formula; black bar) groups. Red signifies higher abundance. No significant group or group × time effect for any of the amino acids identified; linear mixed-effects model.

The concentrations of fecal amino acids over time by formula group are shown in Figure 6. Fecal concentrations of histidine and cysteine were below the limit of detection in 78% and 96% of the samples, respectively, so they were not included in the analyses. There were no significant group or group × time effect identified for any of the remaining amino acids (*p*’s > 0.05).

### 3.4. Increases in the Relative Abundance of Clostridia Related to Leaner Phenotypes

While no measured infant phenotypes differed at baseline (Table 1), the two randomized groups significantly differed in subsequent velocities of weight gain from 0.5 to 4.5 months, in fat mass and percent body fat at 3.5 months, and in WLZ scores at 4.5 months, but did not differ in velocities of linear growth (Table 2). The two groups also significantly differed in relative abundance of Clostridia at both 3.5 and 4.5 months (Figure 1B); we refer to these data, when combined, as occurring at 3–4 months.

**Figure 7:**
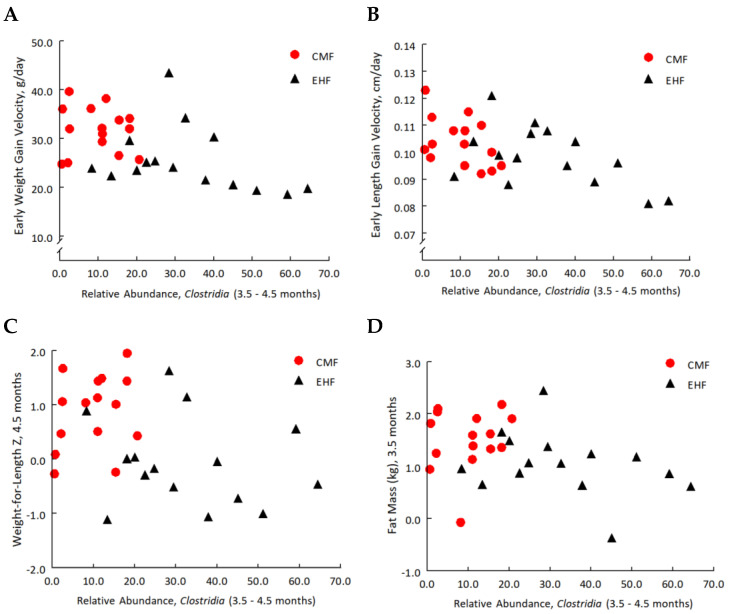
Pearson correlations between relative abundance of *Clostridia* (3–4 months) and (**A**) early weight gain velocity (g/day; *p* = 0.004), (**B**) early length gain velocity (cm/day; *p* = 0.003), (**C**) WLZ at 4.5 months (*p* = 0.02), and (**D**) fat mass (kg) at 3.5 months (*p* = 0.03). Red circles denote CMF (cow milk formula) and black triangles denote EHF (extensive protein hydrolysate) groups.

As shown in Figure 7, the three types of outcomes that differed between the randomized groups—gut Clostridia, velocities of growth and anthropometric phenotypes—were related to each other. The relative abundance of Clostridia at 3–4 months was significantly and inversely correlated with prior weight-gain velocities (Figure 7A; r = −0.51, *p* = 0.004) and length gain velocities (Figure 7B; r = −0.53, *p* = 0.003), WLZ at 4.5 months (r = −0.41; *p* = 0.02; Figure 7C), and fat mass at 3.5 months (r = −0.41; *p* = 0.03; Figure 7D). 

As expected [41,46], greater velocity of weight gain predicted weight status (r = 0.59; *p* < 0.001) and fat mass (r = 0.45; *p* = 0.02) at 1 year. Children who were with overweight at 1 year tended to have lower relative abundance of Clostridia at 3–4 months (15.7 ± 5.3) compared to 1-year-olds who were a healthy weight (27.8 ± 4.2; *p* = 0.08).

## 4. Discussion

While the human gut gradually changes from a predominantly aerobic to anaerobic environment after birth [47,48], in this proof-of-concept study the composition of the gut microbiome underwent striking time-dependent separation based on the type of formula infants were fed, at the age when formula was their sole source of nutrition. Exclusively feeding of EHF, a formula rich in free amino acids, was associated with a more rapid maturation of the infant gut microbiota, with increased alpha diversity dominated by classes of bacteria that degrade carbohydrates (e.g., Lachnospiraceae, Negativicutes, Clostridia). In general, some Veillonella, Lactobacillus, and Streptococcus species within these classes, which are in high relative abundance in the first month [49] and are more commonly associated with the proximal gastrointestinal tract [44], decreased over time, whereas some Bifidobacteria and Clostridium species, which are normally found in a more mature distal gastrointestinal tract community, increased over time [44,45]. However, there were two notable exceptions. 

While *Streptococcus thermophilus* steadily decreased over time in the EHF group, its relative abundance remained high in the CMF group. Conversely, a number of Clostridia species decreased over time in the CMF group, as expected, but significantly increased over time in the EHF group. Indeed, of the seven taxa that exhibited group-dependent or group × time dependent changes, Streptococcus accounted for one taxa while Clostridia accounted for six, including taxa that are associated with the induction of immune tolerance in murine model systems [50,51], that utilize proteins and free amino acids as primary nutrient sources [52,53], or that produce a large array of metabolites by utilizing simple and complex carbohydrates [54]. The carbohydrate-fermentative species *R. gnavus* [55] exhibited the greatest increase in the EHF group over time and its outgrowth distinguished the two infant formula groups approximately 3 months after infants began feeding their assigned formulas. 

These two key compositional differences between CMF and EHF formed the basis of our hypothesis on how feeding CMF or EHF shaped the microbiota in different ways (i.e., increased abundance of Streptococcus among CMF infants and increased abundance of Clostridia among EHF infants). These differences may be acting alone or in concert in causing the observed effects on the infants’ gut microbiota. First, the formulas differ in the form of the protein. The free amino acid concentration is substantially higher in EHF (>9200%) than CMF [18]. Because EHF transits the gastrointestinal tract at a faster rate [56], there may be increased concentrations of amino acids in the colon, where they act as substrates for colonic bacterial growth [57]. Certain Clostridia species may have bioenergetic growth advantage when infants feed EHF because of Strickland fermentation—the coupled oxidation and reduction of amino acids to organic acids—a well-known reaction in Clostridia [58]. However, despite the drastic differences in free amino acids between the infant formulas, the overall concentrations of amino acids in their feces were similar between the two groups, suggesting that, in addition to Strickland fermentation by the gut microbiota, they also likely are being absorbed in the small intestine of the EHF-fed infants. 

Second, differences in carbohydrate source between the infant formulas and, in turn, the availability of carbon sources for metabolism may contribute to group differences in bacterial taxa. Unlike CMF, in EHF one of the carbohydrate sources is modified cornstarch, composed of glucose monomers connected by alpha-1-4 glycosidic linkages and alpha 1–6 glycosidic branch linkages, the latter of which leads to slower enzymatic degradation in the gastrointestinal tract [59]. Not only is the digestion of cornstarch slow in the small intestine, but its fermentation in the colon continues several hours after its ingestion [20]. Indeed, we observed a very robust induction of two Ruminococcus species, *R. gnavus* and *R. torques*, known to be mucinophilic [60,61,62]. Ruminococcus, a genus of bacteria in the Clostridia class, require fermentable carbohydrates for growth [20], and are stimulated by diets high in resistant starches, including cornstarch which contains some resistant starch [20,63]. In addition, *Streptococcus thermophilus,* which was the only taxa that was higher in the microbiota of the CMF than EHF group, has been shown to have increased growth in lactose-containing media compared to media containing the monomers of lactose, namely glucose and galactose [64,65]. In this regard, it is interesting that three of the six bacterial genes showing a significant group by time effect are involved with carbohydrate metabolism [66].

Building on the findings from the entire intent-to-treat cohort of 113 infants [26,41], we found in this subsample of 30 infants that the CMF group had faster weight-gain velocity during the first 4 months, greater fat mass and higher WLZ scores than the EHF group. The present study also revealed that the EHF group had greater abundance of Clostridia class bacteria in the gut than the CMF group, a difference that emerged after feeding the assigned formulas for 3 months. Both of the formula-induced outcomes—relative abundance of Clostridia and clinical phenotypes related to weight —were associated with each other. Greater relative abundance of Clostridia was associated with more normative trajectories of weight and length gain during preceding months and with leaner phenotypes. However, after adjustment for formula treatment group, only the partial correlation with gains in length remained significant. Nevertheless, how fast infants gained weight during the sensitive period of the first 4 months predicted their weight status at one year [67], and overweight 1-year-olds tended to have lower abundance of Clostridia in their guts than those at a healthy weight. Whether or not there is a cause-and-effect relationship between the leaner phenotypes among EHF-fed infants and the greater abundance of will require additional investigation. 

Although the present proof-of-principle study focused on only 30 of the 113 infants enrolled in the RCT and only on fecal samples collected during the first 4.5 months of the year-long trial [26], associations between the abundance of gastrointestinal Clostridia and phenotypes related to obesity have been previously reported in children [68] and adults [69]. Taken together, the data suggest that the formula-induced differences in early weight gain and later weight status [46,70] may be mediated by early priming of the gut microbiota, particularly as it relates to the Clostridia class of bacteria. The impact of Clostridia species on lipid absorption [71] and mucosal immune regulation [50,51] could play a role in the more normative early rapid weight gain among EHF-fed infants [26], which is, in turn, associated with normative weigh status at one year [46]. Additionally, since Clostridia spp. have been shown to enhance immune tolerance in murine models [50], our finding that the consumption of EHF led to increased abundance of certain Clostridia species might be relevant to the observed association between the ingestion of EHF during infancy and the decreased risk for developing atopic disease during childhood and adolescence [72]. Further, a potential energy mechanism for the association between the loss of Clostridia abundance and diversity and obesity and metabolic syndrome comes from T-Myd88-knockout mice [71], increases in Clostridia colonization were associated with a leaner phenotype and down-regulation of CD36, an enterocyte receptor involved in lipid absorption, suggesting decreased energy absorption from the gut. In contrast, lack of colonization with the protective Clostridia in germ-free mice resulted in elevated CD36 expression, weight gain, fatty liver disease, adipose tissue inflammation, and insulin resistance [71]. 

Advantages of this proof-of-principle study include the randomized design, which minimizes selection bias and enables determination of effects of early infant formula diet while keeping other variables constant, thus providing insights into mechanisms of long-term effects of feeding infant formulas that differ in macronutrient composition. While the small sample size and increased likelihood of type II error are limitations, the findings from the proof-of-principle approach hold promise that similarly designed RCTs could serve as a model system for determining mechanisms underlying the divergent clinical outcomes of infants fed different formulas.

In conclusion, diet has strong selective pressure on shaping the composition and functioning of the microbiome [73,74]. What infants are fed is more important than the mode of feeding, since formula-fed infants are not a homogeneous group. Although isocaloric, infant formulas can have different macronutrient composition that have significantly different impacts, in the short term, on infant satiety [75], early weight gain, energy intake, and fecal energy loss [26] and, in the longer term, risks for obesity [21,76] and atopic disease [24]. The present proof-of-principle study revealed distinct compositional changes in the gut microbiota of infants fed formulas differing in the source of and form of protein and carbohydrate for only 3–4 months. The long-lasting consequences of the formula-induced changes to the early bacterial environment as the child transitions to a diet devoid of infant formula remain unknown. Whether the compositional changes in early gut bacterial environment have independent effects on clinical outcomes of growth trajectories, body weight, and fat mass, and whether such changes are biomarkers for later obesity and immune functioning, are important areas of research, for which data are needed from larger, longitudinal RCT cohorts [77] that experimentally manipulate early diet.

## Figures and Tables

**Table 1:** Baseline characteristics of study cohort at study entry (0.5 months).

	Infant Formula Treatment Group		
Characteristics	CMF (*n* = 15)	EHF (*n* = 15)	*p* Value
Age in months	0.39 ± 0.02	0.40 ± 0.02	0.90
Female, *n* (%)	6 (40%)	6 (40%)	1.00
Race/ethnicity, *n* (%)			
Black	9 (60%)	8 (53%)	0.88
White	4 (27%)	4 (27%)	
More than one race/ethnicity	2 (13%)	3 (20%)	
Anthropometry, Z score; 0.5 mos			
Weight for age (WAZ)	−0.54 ± 0.21	−0.37 ± 0.21	0.57
Length for age (LAZ)	−0.73 ± 0.27	−0.65 ± 0.67	0.85
Weight for length (WLZ)	−0.25 ± 0.24	−0.06 ± 0.24	0.59
Body composition ^1^, 0.75 mos			
Fat mass (kg)	0.43 ± 0.08	0.49 ± 0.08	0.60
Percent body fat (%)	11.6 ± 2.0	12.8 ± 2.2	0.70

Mean ± standard error of the mean (SEM) or *n* (%). *n* = 30 unless otherwise indicated; *p* values for main effect of infant formula treatment group (CMF, cow milk formula; EHF, extensive protein hydrolysate formula). ^1^ CMF, *n* = 15, EHF, *n* = 12.

**Table 2:** Growth and body composition in study cohort at 3.5–4.5 months.

	Infant Formula Treatment Group		
Characteristics	CMF (*n* = 15)	EHF (*n* = 15)	*p* Value
Anthropometry, Z scores; 4.5 months			
Weight for age (WAZ)	0.30 ± 0.23	−0.55 ± 0.23	0.02
Length for age (LAZ)	−0.47 ± 0.27	−0.66 ± 0.27	0.61
Weight for length (WLZ)	0.88 ± 0.20	−0.07 ± 0.20	<0.001
Weight-gain velocity (g/day), 0.5–4.5 months	31.77 ± 1.49	25.49 ± 1.49	<0.001
Length-gain velocity (cm/day), 0.5–4.5 months	0.104 ± 0.003	0.094 ± 0.003	0.18
Body composition, 3.5 months ^1^			
Fat mass (kg)	1.61 ± 0.08	1.15 ± 0.12	0.01
Percent body fat (%)	24.3 ± 1.5	19.0 ± 1.5	0.02

Mean ± standard error of mean (SEM). *n* = 30 unless otherwise indicated; *p* values for main effect of infant formula treatment group (CMF, cow milk formula; EHF, extensive protein hydrolysate formula). ^1^ CMF, *n* = 14; EHF, *n* = 14.

## Data Availability

Data described in the manuscript and codebook will be made available upon request pending application.

## Appendix A

**Table A1:** Bacterial taxa (N = 47) with significant time effects ^1^.

Species Name	Time, β Coefficient	Time, *p*-Value
*Actinomyces odontolyticus*	0.12	3.4 × 10^−5^
*Akkermansia muciniphila*	0.28	3.1 × 10^−5^
*Anaerostipes, unclassified*	0.25	6.0 × 10^−8^
*Bacteroides uniformis*	0.07	0.02
*Bifidobacterium bifidum*	0.35	1.0 × 10^−5^
*Bifidobacterium breve*	0.26	1.5 × 10^−4^
*Bifidobacterium longum*	0.39	3.7 × 10^−5^
*Bifidobacterium pseudocatenulatum*	0.11	0.02
*Bilophila, unclassified*	0.06	0.01
*Clostridiales bacterium 1 7 47FAA*	0.23	9.0 × 10^−6^
*Clostridium bartlettii*	0.20	0.02
*Clostridium bolteae*	0.22	3.7 × 10^−5^
*Clostridium difficile*	0.28	8.3 × 10^−8^
*Clostridium hathewayi*	0.11	0.03
*Clostridium perfringens*	−0.26	4.5 × 10^−5^
*Clostridium ramosum*	0.36	1.4 × 10^−9^
*Clostridium symbiosum*	0.13	3.7 × 10^−5^
*Collinsella aerofaciens*	0.22	4.4 × 10^−4^
*Coprobacillus, unclassified*	0.43	5.4 × 10^−13^
*Eggerthella, unclassified*	0.12	7.8 × 10^−5^
*Enterococcus avium*	0.11	1.6 × 10^−5^
*Enterococcus faecalis*	−0.12	0.02
*Erysipelotrichaceae bacterium 21 3*	0.14	3.6 × 10^−5^
*Erysipelotrichaceae bacterium 2 2 44A*	0.12	3.0 × 10^−3^
*Erysipelotrichaceae bacterium 6 1 45*	0.09	0.02
*Eubacterium limosum*	0.06	0.01
*Granulicatella, unclassified*	0.08	1.2 × 10^−3^
*Klebsiella pneumoniae*	0.09	0.01
*Lachnospiraceae bacterium 2 1 58FAA*	0.23	6.0 × 10^−8^
*Lachnospiraceae bacterium 7 1 58FAA*	0.06	0.01
*Lachnospiraceae bacterium 9 1 43BFAA*	0.10	1.3 × 10^−5^
*Lactobacillus fermentum*	−0.13	13.5 × 10^−4^
*Lactobacillus gasseri*	−0.24	3.3 × 10^−5^
*Lactococcus lactis*	0.19	3.8 × 10^−3^
*Megasphaera, unclassified*	0.19	0.04
*Parabacteroides distasonis*	0.58	0.03
*Solobacterium moorei*	0.06	7.1 × 10^−5^
*Staphylococcus hominis*	−0.11	1.2 × 10^−4^
*Streptococcus mitis oralis pneumoniae*	0.08	0.01
*Streptococcus peroris*	0.09	0.01
*Streptococcus salivarius*	−0.33	8.3 × 10^−8^
*Streptococcus vestibularis*	−0.41	8.3 × 10^−8^
*Subdoligranulum, unclassified*	0.16	9.9 × 10^−6^
*Veillonella atypica*	−0.18	1.2 × 10^−4^
*Veillonella parvula*	−0.13	0.02
*Veillonella ratti*	0.30	4.3 × 10^−4^
*Veillonella, unclassified*	−0.15	0.01

^1^ Linear mixed models determined the effect of treatment group (CMF (cow milk formula), EHF (extensive protein hydrolysate formula)), time (0.5, 0.75, 1.5, 2.5, 3.5, 4.5 months), and group × time interaction on fecal bacteria taxa. Table lists the 47 taxa that had only significant time effects.
